# Diversity lost: are all Holarctic large mammal species just relict populations?

**DOI:** 10.1186/1741-7007-8-46

**Published:** 2010-04-21

**Authors:** Michael Hofreiter, Ian Barnes

**Affiliations:** 1 Department of Biology, University of York, York, YO10 5DD, UK; 2 School of Biological Sciences, Royal Holloway, University of London, Egham, Surrey, TW20 0EX, UK

## Abstract

Population genetic analyses of Eurasian wolves published recently in *BMC Evolutionary Biology *suggest that a major genetic turnover took place in Eurasian wolves after the Pleistocene. These results add to the growing evidence that large mammal species surviving the late Pleistocene extinctions nevertheless lost a large share of their genetic diversity.

See research article http://www.biomedcentral.com/1471-2148/10/104

## 

Unraveling the population history of a species is a tricky business. The only archives left are the fossil record, and the traces that population processes have left in the species' gene pool. Both have been fundamental in developing our understanding of how populations existed in the past, but both are ultimately unsatisfying when we come to address how organisms deal - in the long term - with change.

Fossils are prone to the vagaries of taphonomy (the processes by which fossils form), with few individuals even entering the record, and only a small fraction of those surviving and being recovered and analyzed. More problematically, few organisms leave sufficiently robust and informative fossils from which we can readily identify population-level differences. Genetic analyses, on the other hand, are perfectly capable of providing that level of resolution. But genetic data are difficult to interpret. A given gene genealogy may provide little information about past processes or events. Even if the data can be shown to be informative, it may prove impossible to probe in any sort of depth into the past, as populations can readily become admixed, bottlenecked, translocated, or worse still, just flat-out extinct.

The analysis of ancient DNA has done much to bring these two fields of research together. Having struggled in the limelight for much of the 1990s, first as an answer looking for a question, then latterly with some significant issues about data reproducibility, ancient DNA seems more recently to have found itself very much at home in facilitating comparisons between current and past populations, with a timescale of around the past 50,000 to 100,000 years. Conveniently situated in the midst of this period is a suite of large mammal extinctions and range changes. Even better, the megafaunal extinctions that began at the end of the Pleistocene (from around 50,000 years ago) are well studied, have a well-established chronology (due to the ready availability of radiocarbon dating) and remain controversial, with the relative roles of a globally expanding, top predator (*Homo sapiens*) and a complex series of climate shifts yet to be convincingly resolved.

In this context, one question that ancient DNA can readily address is the extent to which species surviving the Late Pleistocene extinctions experienced population loss or replacement. While we can be quite certain that extinct species experienced reductions both in population size and genetic diversity, it is much less clear to what extent the environmental and ecological turmoil of the Late Pleistocene affected those species that survive to the present day.

Therefore, comparisons between the genetic diversity of past and present populations of a species, such as the study on Eurasian wolves (*Canis lupus*, Figure 1) recently reported in *BMC Evolutionary Biology *by Pilot *et al. *[1], are of great value for understanding the impact these climatic and environmental changes had on surviving species. In a pioneering study addressing this issue, Shapiro *et al. *[2] showed that the genetic diversity of modern American buffalos (*Bison bison*) is a tiny fraction of what it had been during the Pleistocene. A more recent study on American wolves demonstrated a complete replacement of one haplogroup (a set of related DNA sequences for a specific locus, in this case, as usually, mitochondrial DNA (mtDNA)) by a second one some 11,000 years ago [3]. Using published ancient DNA sequences and both published and newly generated sequences from modern wolves, Pilot *et al. *[1] have now taken this analysis over to Eurasia. They found that while haplogroup 1, which disappeared from North America, still exists in Eurasia, it was much more common during the Pleistocene and Holocene than it is today. Moreover, haplogroup 2, which completely replaced haplogroup 1 in North America, also became much more common in Europe. Analyses of stable isotopes, which allow conclusions about the diet, and therefore ecology, of the extinct wolf populations, furthermore suggest that the Pleistocene wolves from haplogroup 1 mainly preyed on Pleistocene megafaunal species [3,4], which became rare at the beginning of the Holocene 12,000 years ago.

**Figure 1:**
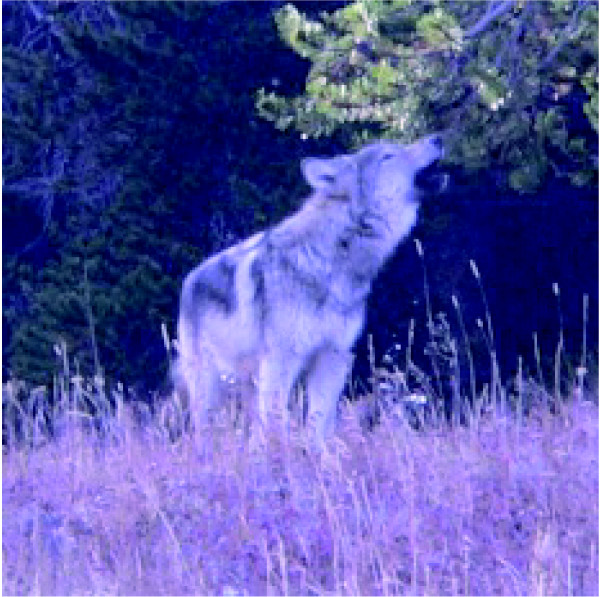
**Howling wolf**. A recent population genetic study on Eurasian wolves showed that they experienced a major turnover in genetic diversity during the Holocene. Picture courtesy of Bob Wayne.

## Diversity losses inferred from ancient DNA

Although the analysis of ancient DNA is still a tedious process, with very limited survival of DNA molecules in fossils, studies of ancient DNA have already revealed a lot about changes in the genetic makeup of populations over time. Whereas analyses of modern DNA invariably miss events, such as haplogroup extinctions, that took place in the past, ancient DNA analyses have the potential to achieve precisely this.

The first study to demonstrate the complexity of past population history was published in 2002 on brown bears (*Ursus arctos*) from Alaska and Canada [5]. In this study, it was possible to identify a haplogroup extinction event in Alaska around 35,000 years ago, with subsequent replacement some 15,000 years later by bears carrying the haplogroups that still exist in the region today. Subsequent studies on European brown bears also later documented a substantial loss of genetic diversity before and during the Holocene [6,7]. In fact, every study so far that has compared the genetic diversity of Pleistocene populations to their modern counterparts has detected a considerable loss of genetic diversity. For species such as muskox (*Ovibos moschatus*), bison or spotted hyena (*Crocuta crocuta*), for which Eurasian populations disappeared and the species only survived in North America (muskox and bison) and Africa (spotted hyena), this may not come as a complete surprise [2,8,9]. However, even if the picture is restricted to areas in which species are continuously present, losses of genetic diversity are obvious, such as for North American wolves and bison as well as for European brown bears.

## Ecological factors in diversity loss

So far, we know very little about the potential factors that drive reduction in genetic diversity for the extant large mammals of the northern continents - the Holarctic. It does not help that the causes of megafaunal extinctions at the end of the Pleistocene are also still unresolved in that region [10]. In part this results from the difficulties of investigating subtle ecological differences between populations of the same species through fossil remains. Again, the wolves lead the way with respect to these questions, as we now have not only genetic data, but also isotopic signals and - for North American individuals - tooth-wear patterns, both of which are informative with regard to the prey spectrum. These data suggest that both Eurasian and North American Pleistocene wolves preyed on megafaunal species, specifically horses and large bovids. According to Pilot *et al. *[1] this fact may also offer an explanation as to why replacement was only partial in Europe but complete in North America, as horses, one of the most important prey species of Pleistocene wolves, went extinct in North America but survived in Europe. Interestingly, not only the timing of extinction of horses in North America [11] but also the minimum population size for North American bison [2] coincide surprisingly well with the extinction of wolf haplogroup 1 in North America, supporting the notion that the disappearance of their prey caused the extinction of this wolf ecomorph (a morphological type specifically adapted to particular ecological conditions, such as a particular prey species).

It may come as a surprise that these wolves were neither able to adapt to a new prey spectrum nor did their mtDNA sequences survive as a result of gene flow with neighboring or incoming wolves. However, although wolves and carnivores in general are usually highly mobile, ecological factors, including specializations on certain prey, seem to play an important role in shaping the geographical structure of genetic diversity in extant wolf populations [12,13]. Therefore, large-scale ecological changes may invariably result in losses not only of specialized ecomorphs but also of the genetic diversity they carry.

## Losing ecomorphological and genetic diversity

The observed losses of genetic and ecomorphological diversity are not restricted to carnivores, as similar observations have been made for bison. Not only did the species as a whole lose a large part of its genetic diversity, modern bison are also surprisingly homogeneous in their morphology when compared to their Pleistocene counterparts, which were diverse enough to have been placed into multiple species. The same seems to be true for horses, the Pleistocene representatives of which have also been divided into numerous species. However, a recent ancient DNA study has suggested that the morphological range of extinct horse species was much larger than has been assumed taking modern horses as a baseline [14].

It therefore seems that extant species - at least in the northern Holarctic - are depleted in both genetic and ecomorphological diversity when compared with their Pleistocene counterparts. Given that the last glaciation ended only some 10,000 years ago, this is not entirely surprising. If we assume that diversity is lost during glacial maxima and builds up between them, then we would expect Late Pleistocene populations to show more diversity in both respects, as they had almost 100,000 years to accumulate it. The question is what lessons we can draw from this?

## Biodiversity during the Pleistocene, today and in the future

Compared with the Late Pleistocene, we have undoubtedly lost a lot of biodiversity, and it seems unlikely that we will develop the technology to bring it back in the near future. However, studies like that by Pilot *et al. *[1] are starting to reveal that at least some surviving species are also depleted in genetic diversity compared with their Pleistocene ancestors. Although we have ancient DNA data for only a few extant species, as researchers have tended to focus on the extinct ones, DNA sequences from modern populations suggest that many living species may have much lower genetic diversity than they had during the Pleistocene. Yet despite this, many species were extremely successful during the Holocene until technological advances allowed humans to severely affect their population size; once again, American bison are an obvious example.

Converting this observation into conservation policy requires a more extensive discussion between some apparently quite disparate members of the scientific community - paleontologists, conservation biologists and ancient DNA specialists among others. One possibility is that the best way to preserve biodiversity for the future is not to try to maximize mitochondrial sequence diversity in a population, but rather to take an active role in encouraging species to colonize as broad a habitat as naturally possible, drawing from the Late Pleistocene records. Although none of us will live long enough to witness the result, such a strategy could allow species not only to build up genetic diversity but also to again reach the ecomorphological variability so aptly demonstrated by the fossil record.
